# Obstructive Sleep Apnea after COVID-19: An Observational Study

**DOI:** 10.3390/life14081052

**Published:** 2024-08-22

**Authors:** George-Cosmin Popovici, Costinela-Valerica Georgescu, Mihaela-Camelia Vasile, Constantin-Marinel Vlase, Anca-Adriana Arbune, Manuela Arbune

**Affiliations:** 1 School for Doctoral Studies in Biomedical Sciences, “Dunarea de Jos” University from Galati, 800008 Galati, Romania; popvicigeorge1989@gmail.com (G.-C.P.); mihaela.vasile@ugal.ro (M.-C.V.); constantin.vlase@ugal.ro (C.-M.V.); 2 Pneumophtiziology Hospital Galati, 800189 Galati, Romania; 3 Pharmaceutical Sciences Department, “Dunarea de Jos” University from Galati, 800008 Galati, Romania; 4 Gynecology and Obstetrics Clinic Hospital Galati, 544886 Galati, Romania; 5 Clinic Hospital for Infectious Diseases Galati, Department II, 800179 Galati, Romania; 6 Military Hospital “Dr. Aristide Serfioti” Galati, 800150 Galati, Romania; 7 Neurology Department Clinic Institute Fundeni Bucharest, 022328 Bucharest, Romania; 8 Medical Clinic Department, “Dunarea de Jos” University from Galati, 800008 Galati, Romania; manuela.arbune@ugal.ro; 9 Clinic Hospital for Infectious Diseases Galati, Department I, 800179 Galati, Romania

**Keywords:** COVID-19, sleep apnea, polygraphy

## Abstract

The risk factors of hospitalized COVID-19 and obstructive sleep apnea (OSA) overlap. The aim of this study is to evaluate the prevalence and associated factors of post-COVID-19 OSA in hospitalized adult patients from southeastern Romania. A follow-up study was conducted on patients hospitalized for COVID-19 at the Pneumology Hospital in Galati, Romania, between 2021 and 2022. OSA was evaluated using the Epworth and STOP-BANG questionnaires and nocturnal polygraphy monitoring. Out of 331 patients, 257 were evaluated for sleep apnea in the 12th week. The prevalence of severe OSA was 57.97%. Significant associations were found with male gender, an age over 60, obesity, and cardiovascular co-morbidities. Non-invasive ventilatory therapy (NIV) and a hygienic–dietary regimen were recommended based on severity following a control visit after a month. Developing strategies for diagnosing and monitoring sleep disorders, including home sleep apnea tests and patient education, are the next directions for post-COVID-19 management.

## 1. Introduction

Obstructive sleep apnea (OSA) syndrome is characterized by the occurrence of at least five respiratory events (apneas and hypopneas) per hour of sleep. An apnea event is defined as a complete cessation of respiratory flow (both nasal and oral) lasting for at least 10 s, whereas hypopnea is defined by a reduction in respiratory flow by more than 30–50% accompanied by a decrease in blood oxygen saturation [1].

Sleep apnea syndrome is classified into three types: obstructive (OSA), central (CSA), and mixed (MSA). The most common type is OSA, which is characterized by the cessation of respiratory flow with the persistence of thoraco-abdominal respiratory movements. The central type (CSA) is characterized by the cessation of airflow and the absence of ventilatory effort, and is generally associated with cardiovascular or neurological diseases. The mixed type (MSA) presents both central and obstructive respiratory events [2].

The prevalence of mild forms of OSA in the general population ranges between 9% and 38%, while that of moderate and severe forms ranges between 6% and 17%, with an increasing trend in recent years [3,4]. Smoking, diabetes mellitus, hypertension, asthma, and obesity increase susceptibility to developing OSA [5,6].

Symptoms of obstructive sleep apnea syndrome (OSA) include loud and irregular snoring, sleep disturbances, morning headaches, and excessive daytime sleepiness (EDS). Consequently, work capacity, intellectual ability, and concentration decrease, and memory and personality disorders may appear. These manifestations are often accompanied by irritability and depression, predisposing people to family conflicts, work conflicts, and traffic accidents [7,8]. 

The COVID-19 pandemic, declared by the World Health Organization in 2020, showed that patients with OSA have an eight-fold increase in the risk of presenting symptomatic forms of this coronavirus infection as compared to non-OSA patients, as well as a higher risk of severe evolution and death [9,10]. 

The mechanisms by which OSA increases the severity of COVID-19 may include the exacerbation of endothelial dysfunction, inflammation, oxidative stress, or lung injuries, overlapping with risk factors for OSA [5,11]. 

The risk of death from COVID-19 is increased by the presence of hypertension, diabetes mellitus, obesity, heart, and cerebrovascular diseases [12,13,14]. Conversely, COVID-19 may increase the risk of developing OSA [15]. 

Post-acute COVID-19 symptoms such as fatigue, dyspnea, chest pain, daytime sleepiness, memory, and concentration disorders can persist for more than 12 weeks in long COVID syndrome. Sleep apnea and decreased sleep efficiency may be treatable causes of some of these manifestations if early diagnosis and specific OSA interventions are provided [16]. 

We aimed to evaluate post-COVID-19 sleep disorders in hospitalized adult patients from southeastern Romania. 

## 2. Materials and Methods

We conducted an observational study on sleep respiratory disorders in patients hospitalized for COVID-19 in a department of the Pneumology Hospital in Galati, Romania between 2021 and 2022. Giving the local pandemic medical regulations during the study, the hospital was “COVID-only”. However, the critical patients couldn’t be cared in our hospital because an intensive care unit was not available. The baseline evaluation of sleep disorder was conducted after 12 weeks following COVID-19 hospitalization. The characteristic data of COVID-19 outcomes were retrospectively acquired from the hospital’s database. The diagnosis and treatment of COVID-19 were conducted according to the national protocol for this infection [17]. 

Participants were over 20 years of age, had no history of OSA as provided by the patient, and gave informed consent for inclusion in this study. The treatment was based mainly on Automatic Continuous Positive Airway Pressure (APAP), but Bi-Level Positive Airway Pressure (BiPAP) was used in some severe cases. Diet and sleep hygiene recommendations were provided for all the patients. A follow-up evaluation was conducted one month post-treatment. 

Post-COVID-19 evaluation was based on the Epworth and STOP-BANG questionnaires, as well as nocturnal polygraphic monitoring [18]. Patients completed the two screening questionnaires. The STOP-BANG questionnaire for obstructive sleep apnea (OSA) risk was scored from 0 to 8, with 1 point for each of the following identified risk factors: snoring, tired, observed apnea, pressure (hypertension), high body mass index, age (over 50 years), neck circumference (over 43 cm), and gender (male). A high risk for apnea is represented by a score of 5–8, moderate risk by a score of 3–4, and low risk by a score below 2 [19]. The Epworth Sleepiness Scale (ESS) assessed the risk of dozing in eight situations, scoring from 0 to 3 points for each circumstance. The maximum score is 24, and a score above 10 indicates pathological sleepiness [20]. Polygraphic recording was performed on an outpatient basis, without medical supervision (type II), using the VitalNight Plus “LÖWENSTEIN” cardio-respiratory polygraph and VitalNight software. Preparation for polygraphic sleep test (PG) required the absence of antidepressant medication, MAO inhibitors, alcohol, and caffeine. The 12 channels of the polygraph recorded signals for the following physiological variables: airflow, snoring, body position during sleep, ambient light, oxygen saturation (SpO2), pulse frequency, pulse wave, positive airway pressure titration, thoracic effort, abdominal effort, respiratory rate, and respiratory effort (the phase shift between thorax and abdomen). The duration of nocturnal recordings was 8 h. Interpretation was performed according to the American Academy of Sleep Medicine (AASM) Manual for the Scoring of Sleep and Associated Events [21]. The apnea–hypopnea index (AHI) was calculated as the number of apneas and hypopneas per hour of sleep. The AHI value classified OSA cases by severity: mild forms (5–15), moderate (15–30), and severe (>30) [22,23]. Adherence to therapy was measured by analyzing data recorded on the compliance card of the non-invasive ventilation device during the first month of treatment. Adherence was considered to be satisfactory when the device was used for at least five hours per night on at least 70% of the evaluated days [24]. This study was performed in accordance with the Declaration of Helsinki and received institutional approval from the Ethics Committee of the Pneumology Hospital Galati (under the number 225/09.01.2024).

Statistical analysis used the XLSTAT program version 2022.4.5. Descriptive statistical evaluation and correlation analysis were performed. Quantitative variables were described with the mean ± standard deviation, and qualitative variables were described with numbers and percentages. Normal distribution was assessed using histograms, observing the frequency distribution of quantitative data. Continuous variables were compared using Student’s *t*-test, and proportions were compared using the chi-square test or Fisher exact test. Bivariate analysis of the association of severe forms of OSA with demographic data, co-morbidities, or severe prognostic factors for COVID-19 used logistic regression. One-way multivariate analysis of variance (MANOVA) was conducted to determine whether there is a difference in sleep parameters considering different co-morbidities, COVID-19 vaccination status, age, or sex. Adjusted partial eta squared (adj η^2^_p_) was used to measure effect size. All *p*-values < 0.05 were considered statistically significant.

## 3. Results

Out of 331 hospitalized patients with COVID-19, 257 received follow-ups for sleep apnea in the 12th week. The patients eligible for NIV received the treatment recommendation and a control visit was planned after one month of treatment.

### 3.1. Demographic Data

The ages of the evaluated post-COVID-19 patients ranged from 24 to 82 years, with an average age of 57.27 ± 11.33 years. The majority were male (66.5%), resided in urban areas (64.2%), possessed secondary education (51%), were non-smokers (57.2%), and reported abstaining from alcohol consumption (70.82%) [Table 1]. 

### 3.2. Co-Morbidities Associated with Post-COVID-19 OSA

**Figure 1:**
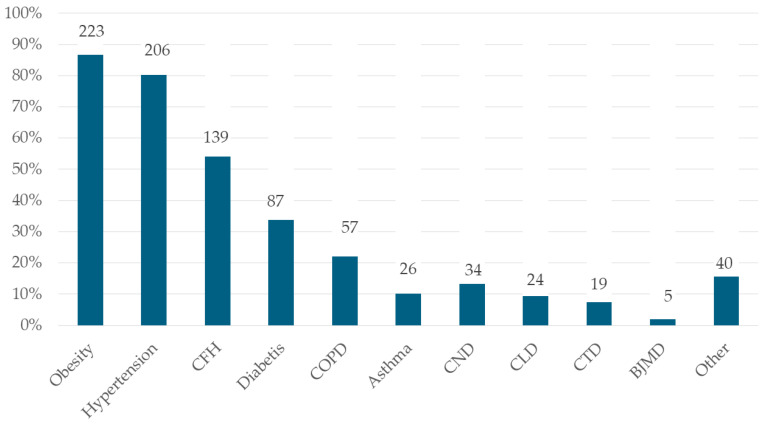
Frequencies of co-morbidities associated with COVID-19. Legend: CHF—chronic heart failure; COPD—chronic obstructive pulmonary disease; CND—chronic neurologic disease; CLD—chronic liver disease; CTD—connective tissue diseases; BJMD—bone, joint, muscle diseases.

The most frequent co-morbidities were obesity (87.77%), hypertension (80.15%), chronic heart failure (54.08%), diabetes (33.85%), and chronic obstructive pulmonary diseases (22.17%) [Figure 1].

### 3.3. Clinical, Biological, and Radiological Characteristics of the COVID-19 Episode

Post-COVID-19 OSA evaluation included 57.2% of patients who were vaccinated against COVID-19, with 39.7% treated after the first COVID-19 episode and 60.3% after a reinfection episode. In terms of disease severity, 72.4% had mild forms, 25.3% had moderate forms, and 2.3% had severe forms of COVID-19 [Table 2].

### 3.4. OSA Screening Questionnaire

The Epworth Sleepiness Scale (ESS) scores ranged from 1 to 23, with a mean of 14.12 ± 4.14 [Table A1]. Pathological sleepiness, indicated by scores above 10, was found in 86.38% of patients. The highest chance of dozing was reported when sitting quietly after lunch, without alcohol, in 38.52% of patients.

The STOP-BANG questionnaire scores ranged from 0 to 8, with a mean of 4.98 ± 1.62 [Table A2]. The apnea risk was stratified by the questionnaire score, identifying 59.92% as having high risk, 33.07% moderate risk, and 7% low risk. The patient profile contributing most frequently to the high OSA risk is a male, over 50 years old, and hypertensive.

### 3.5. Nocturnal Polygraphy

The apnea–hypopnea index (AHI) quantified the severity of sleep apnea by counting the number of apnea and hypopnea events during sleep. We found AHI values ≥ 5 in all patients, excepting two cases. The distribution of patients by OSA severity, corresponding to AHI, indicates that 58.75% had severe forms (AHI > 30), 29.18% moderate forms (15–30), and 11.28% mild forms (5–15). Central apnea events were present in only 22.56% of cases, but most cases showed obstructive pulmonary forms. The Oxygen Desaturation Index had values above 15/h of sleep in 82.87% of patients, interpreted as highly suggestive for OSA. Oxyhemoglobin desaturation (SpO2 normal 95–100%) was graded in three severity levels: 16.73% severe desaturation (SpO2 < 88%), 13.61% moderate (88–90%), and 16.73% mild (91–94%) [Table 3]. 

**Figure A1:**
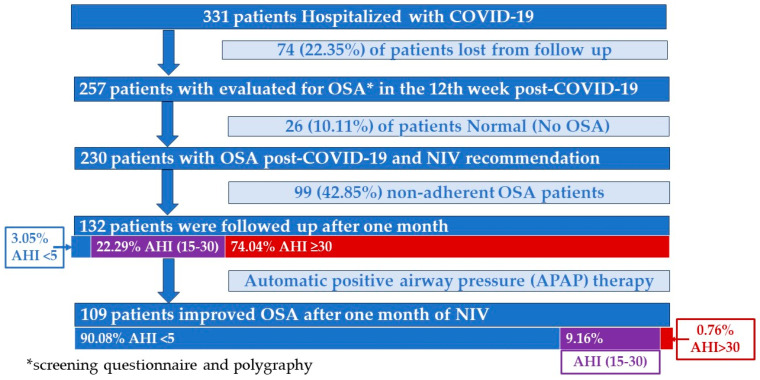
Flow chart of post—COVID-19 interventions for OSA.

Non-invasive ventilation (NIV) was indicated in 230 (89.53%) cases, but only 132 patients followed the recommendations and attended the first month follow-up visit, indicating an adherence rate of 57.39%. The residual AHI decreased in all cases, reaching a mean value of 2.63 ± 2.10. Automatic or Bi-Level positive airway pressure (APAP/BiPAP) therapy achieved the intervention goal of AHI under five per hour in 89.39% of patients. A single case with moderate OSA after APAP was reported in a 66-year-old male smoker (40 packs/year), obese (BMI 39 kg/m^2^) with multiple co-morbidities, where AHI reduced from 66.1 to 15.3 [Figure A1].

### 3.6. Factors Associated with Severe OSA Forms

**Figure 2:**
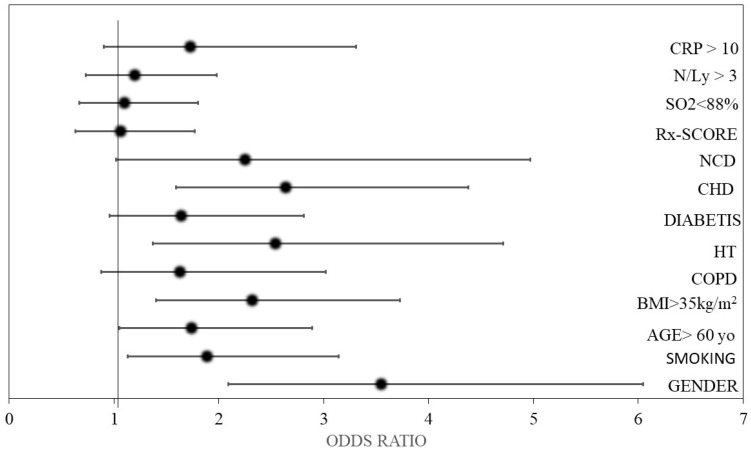
The forest plots for factors associated with severe OSA (AHI ≥ 30). Legend: BMI: body mass index; COPD: chronic obstructive pulmonary disease; CHD: vhronic heart disease; NCD: neurocognitive diseases; Rx: radiologic; N/Ly: neutrophile/lymphocyte ratio; CRP: C-reactive protein.

Severe OSA (AHI > 30) correlated with male gender, class 2–3 obesity, smoking, an age over 60 years, and cardiovascular co-morbidities such as hypertension and chronic heart diseases. However, other known factors with a prognostic role for severe COVID-19 evolution, such as respiratory dysfunction, radiological lesion extent, neutrophil/lymphocyte ratio, and elevated CRP values, did not significantly influence the post-COVID-19 evolution of OSA [Figure 2; Table 4].

### 3.7. Factors Associated with Post-COVID-19 Sleep Parameters

One-way MANOVA was conducted to determine whether there is a difference in sleep parameters considering the associated co-morbidities, but there were no significant results in the entire group. However, we performed further analysis in two separate groups—new infections and reinfections. Specific co-morbidities seem to have a significant impact on specific sleep parameters in the reinfections group. The presence of a chronic heart disease has an effect on the SpO2 time spent under 90%, F(1,50) = 5.524, *p* = 0.023, adj η^2^_p_ = 0.99, and the respective snoring index/h, F(1,50) = 6.122, *p* = 0.017, adj η^2^_p_ = 0.109. The presence of diabetes type 2 has an effect on the total number of hypopneas, F(1,50) = 6.823, *p* = 0.012, adj η^2^_p_ = 0.120, and the respective snoring index/h, F(1,50) = 8.578, *p* = 0.005, adj η^2^_p_ = 0.146. We also found a significant effect of an age over 60 years on the total number of hypopneas, F(1,129) = 10.329, *p* = 0.007, adj η^2^_p_ = 0.055, as well as on the snoring index/h, F(1,129) = 7.444, *p* = 0.002, partial eta squared = 0.074. 

When looking at unvaccinated patients with a one-way MANOVA analyzing the effect of gender on sleep parameters, we identified a significant difference between genders only in the total number of hypopneas, F(1,78) = 4.391, *p* = 0.039, adj η^2^_p_ = 0.053, with significantly higher values in males. Conversely, the analysis of vaccinated patients again identified significantly higher values in males, but in the total number of apneas, F(1,48) = 5.247, *p* = 0.026, adj η^2^_p_ = 0.099. 

## 4. Discussion

COVID-19 and sleep apnea are overlapping health problems, both involving hypoxemia and multisystem distress. While sleep apnea involves gradual reductions in breathing, COVID-19 causes lung injuries with consequent respiratory failure [26]. 

The frequency of severe post-COVID-19 OSA forms in our study was 57.97%, significantly higher than the prevalence in the general population, which is reported to be between 6% and 17%. This finding aligns with previous studies that have reported increased sleep disorders following COVID-19 infection [27,28]. Considering that 75% of OSA cases remain undiagnosed, our study data might reflect the “unveiling” of these conditions in patients who had respiratory involvement during COVID-19. 

Our study found a significantly higher prevalence of severe OSA in post-COVID-19 patients compared to the general population [3]. The high prevalence of severe OSA may be due to previously undiagnosed cases before acute COVID-19 illness. 

Diabetes mellitus, hypertension, asthma, and obesity are factors that increase susceptibility to OSA, as confirmed by our study. On the other hand, OSA, even in undiagnosed forms, is associated with a state of inflammation and cellular immunity dysfunction [29,30]. Intermittent hypoxia in OSA contributes to the release of IL-6 and TNF-α and to increased CRP, potentiating the consequences of pulmonary respiratory failure during COVID-19 infection [31]. 

Compared to the estimated 0.9% incidence of central apnea in the general population, our polysomnographic study identified 22.56% of cases [32]. Central apnea influences morbidity and mortality, especially in patients with concomitant heart failure or stroke and in users of substances that depress respiratory centers. The neuroinvasive potential of SARS-CoV-2 may contribute to the central component of acute respiratory failure, but the relationship with COVID-19 is unclear regarding both newly appeared central apnea and exacerbations of pre-existing central apnea [33]. 

However, the increased frequency of post-COVID-19 central sleep apnea diagnosed by polysomnography could be overestimated given the limitations of current criteria for differentiating central and obstructive hypopnea, the possibility of concomitant occurrence of the two forms of sleep apnea, and the variability of expression depending on associated co-morbidities [34,35]. 

Pre-existing inflammation found in OSA, as well as in obesity, diabetes mellitus, or chronic heart diseases, increases the risk of hospitalized forms of COVID-19, probably explaining the higher likelihood of OSA in patients hospitalized for COVID-19 [36]. 

The study by Lin WC and colleagues on the prevalence of OSA at 3 months post-COVID-19 indicates an equal risk for women and men to develop OSA, unlike the present study, which highlights the correlation with male gender, probably due to the different study design [15]. 

The role of COVID-19 in the onset of OSA is controversial. A large case-control study conducted in 2020 evidenced that sleep-disordered breathing and sleep-related hypoxia were not associated with increased SARS-CoV-2 positivity, but sleep-related hypoxia was an associated risk factor for severe outcomes of the viral disease [26]. In a retrospective study on adult patients diagnosed with COVID-19 prior to the Omicron wave, the diagnosis of obstructive sleep apnea was found to be an independent risk factor for severe viral disease but not for increased mortality [37]. 

Sleep disorders are common manifestations of the “post-COVID-19” syndrome, defined as chronic manifestations associated with infection present at least 3 months after COVID-19, with continuous progressive evolution or with relapses and remissions, affecting one or more body systems [38]. Treating sleep disorders can contribute to their improvement or remission, as evidenced by the favorable outcomes of NIV therapy in our study’s compliant OSA patients. The use of accessible screening tools, such as the STOP-BANG questionnaire or the Epworth Sleepiness Scale score, is very useful for evaluating post-COVID-19 patients, especially those with additional risk factors. Management of post-COVID-19 patients should be performed by multidisciplinary medical teams, including sleep medicine specialists, with the capacity for complex patient care and recovery, as well as patient education and research [39].

According to the results of our study, outcomes of NIV therapy in compliant patients with OSA were favorable. The clinical implications of these findings are substantial. Given the association between OSA and severe COVID-19 outcomes, it is crucial to implement routine screening for OSA in post-COVID-19 patients, particularly for those with risk factors such as obesity, hypertension, and older age. Telemedicine and home sleep apnea tests could play a significant role in this context, offering a practical approach to diagnose and manage OSA remotely [40]. 

There are no published studies addressing the relationship between COVID-19 reinfection or vaccination and sleep parameters. Our data bring new insights into this relationship. However, there is evidence suggesting that patients with pre-existing obesity or chronic heart diseases have a higher risk of post-COVID-19 complications related to individual immune and pro-inflammatory pathophysiologic mechanisms [41]. Post-COVID-19 conditions, including sleep disorders and cardiac symptoms, are influenced by age, gender, and pre-existing sleep disturbances [42]. 

The relation between the total number of hypopneas in patients with a new infection and the number of recorded apneas in vaccinated males was not considered in studies until now. We hypothesize that an active COVID-19 infection can unmask hypopneas in unvaccinated patients, while in vaccinated patients apneas become more evident. This result could be supported by the association of central apnea in mixed forms, possibly related to respiratory disfunction and brain hypoxia during severe acute viral disease [43,44]. Future comprehensive research is required to understand the relationship between sleep disorders and COVID-19. 

*Limitations of the study:* The reliance on self-reported data for some variables may introduce bias. The retrospectively acquired data during COVID-19 hospitalization could be less accurate. A sleep study was not available before COVID-19 and we could not be sure that patients had OSA before the acute infection. We have no control group because during the study time, the hospital was “COVID-only”. New OSA diagnoses were based on the patients’ medical history. A screening questionnaire was not available upon the patients’ hospitalization for COVID-19. 

This study should have conducted longer follow-up post-COVID-19 OSA evaluations, including a higher number of patients and multicenter studies. Future research should focus on multicenter studies and explore the long-term outcomes of post-COVID-19 OSA in different populations.

## 5. Conclusions

Post-COVID-19 OSA is prevalent in patients with specific risk factors. Predictors identified for severe post-COVID-19 OSA forms correspond to traditional risk factors known for OSA in the general population, such as male gender, an age over 60 years, smoking, and co-morbidities (obesity, chronic cardiovascular, and neurological diseases). 

The lack of correlation between OSA severity and COVID-19 prognostic markers suggests that COVID-19 may act as a triggering factor for OSA or exacerbate pre-existing undiagnosed conditions, rather than having a causal relationship. 

Developing monitoring strategies for post-COVID-19 patients that include sleep disorder screening, the use of non-invasive therapies in diagnosed cases, and patient education for adherence can contribute to improving the prognosis of patients with post-COVID-19 syndrome.

## Figures and Tables

**Table 1:** Demographic characteristics of patients with post-COVID-19 evaluation for OSA.

N = 257		n	%
Sex	Male	171	66.5%
	Female	86	33.5%
Living	Urban	165	64.2%
	Rural	92	35.8%
Education	≤4 years	5	1.9%
	8 years	131	51.0%
	≥12 years	121	47.1%
Professional	Employed	135	52.5%
	Unemployed	10	3.9%
	Retired	112	43.6%
Smoker	Yes	110	42.8%
	No	147	57.2%
Alcohol	Yes	75	29.18%
	No	182	70.82%

**Table 2:** Characteristics of COVID-19 episodes before OSA evaluation.

N = 257	n	%	*p*	CI 0.95 for π
COVID-19-vaccinated	110	42.8%	0.020	0.36; 0.48
COVID-19 history (Reinfection)	155	60.3%	<0.001	0.54; 0.66
Severe COVID-19	186	72.4%	<0.001	0.68; 0.77
Fever	130	50.58%	0.851	0.44; 0.56
Headache	157	61.09%	<0.001	0.55; 0.67
Symptoms > 4 days	110	42.8%	0.020	0.36; 0.48
Anosmia	54	21.01%	<0.001	0.16; 0.26
Heart rate > 100/min	143	55.64%	0.070	0.49; 0.61
Respiratory rate > 24	53	20.62%	<0.001	0.15; 0.25
SO2 < 88%	118	45.91%	0.190	0.39; 0.52
Rx-score > 4	94	36.58%	<0.001	0.30; 0.42
N/Ly > 3	139	54.09%	0.190	0.47; 0.60
CRP > 10	50	19.46%	<0.001	0.75; 0.85
Na < 136 mEq/L	152	59.38%	0.002	0.53; 0.65
ALT > x1.5 NV	140	54.47%	0.151	0.68; 0.60
Cortico-therapy	67	20.07%	<0.001	0.20; 0.31
Anticoagulant	70	27.24%	<0.001	0.21; 0.32

**Table 3:** Characteristics of PG evaluation in post-COVID-19 patients.

	Average ± SD	Median	Min	Max
Apnoea–hypopnea index	37.55 ± 22.81	32	1.9	106.5
Apnea Index	171.88 ± 172.51	103	2	728
Central apnea	14.5 ± 36.31	2	0	316
Hypopnea Index	97.26 ± 60.46	89	4	296
Snoring/Sleeping time Index	108.96 ± 145.98	45	0	745.6
Oxygen Desaturation Index	37.86 ± 23.15	34.4	2.3	100.7
Average saturation at night	90.6 ± 4.4	92	72	98
T90	24.63 ± 28.74	12.9	0	99.6

Legend: T90—proportion under 90% oxygen saturation.

**Table 4:** Correlation analysis between severe OSA forms and severe prognostic factors for COVID-19.

		AHI ≥ 30	AHI < 30	or	*p*	CI
SEX	M	116	55	3.55	<0.001	2.09; 6.05
	F	32	54			
SMOKING	YES	73	37	1.894	0.013	1.13; 3.14
	NO	75	72			
AGE > 60	YES	73	75	1.74	0.030	1.05; 2.89
	NO	39	70			
BMI > 35 kg/m^2^	YES	97	49	2.32	<0.001	1.40; 3.73
	NO	51	60			
COPD	YES	38	19	1.63	0.115	0.88; 3.02
	NO	110	90			
HYPERTENSION	YES	128	78	2.54	0.003	1.37; 4.71
	NO	20	31			
DIABETES	YES	57	91	1.64	0.065	0.96; 2.81
	NO	30	79			
CHD	YES	95	44	2.64	<0.001	1.59; 4.38
	NO	53	65			
NCD	YES	25	9	2.25	0.043	1.02; 4.97
	NO	123	100			
Rx-score > 4	YES	55	39	1.06	0.746	0.63; 1.77
	NO	93	70			
SpO2 < 88%	YES	69	49	1.10	0.703	0.67; 1.80
	NO	78	61			
BPM > 22	YES	46	33	1.06	0.824	0.62; 1.81
	NO	101	77			
N/Ly > 3	YES	83	56	1.20	0.454	0.73; 1.98
	NO	65	53			
CRP > 10 mg/dL	YES	34	16	1.73	0.096	0.90; 3.31
	NO	114	93			

Legend: AHI: apnea–hypopnea index [21,22,23]; BMI: body mass index; COPD: chronic obstructive pulmonary disease; CHD: chronic heart disease; NCD: neurocognitive diseases; Rx-score: radiologic score [25]; BPM: breaths per minute; N/Ly: neutrophile/Lymphocyte ratio; CRP: C-reactive protein.

## Data Availability

The raw data supporting the conclusions of this article will be made available by the authors on request.

## Appendix A

**Table A1:** Scores of Epworth Sleepiness Scale (ESS) in post-COVID-19 patients.

Situation	0	1	2	3	Median Score	Average Score ± SD
Sitting and reading	3	42	121	91	2	2.16 +/− 0.73
Watching TV	8	86	120	86	2	1.77 +/− 0.75
Sitting inactive in a public place	7	83	143	24	2	1.71 +/− 0.66
Riding in a car for an hour without break	2	79	138	38	2	1.82 +/− 0.67
Lying down to rest in the afternoon	5	160	67	25	1	1.43 +/− 0.69
Sitting talking to someone	11	163	82	0	1	1.28 +/− 0.53
Sitting quietly after lunch, without alcohol	1	64	93	99	2	2.12 +/− 0.79
Stopped in traffic for a few minutes	19	66	96	76	2	1.89 +/− 0.92

Legend: 0 = No chance of dozing; 1 = slight chance of dozing; 2 = Moderate chance of dozing; 3 = High chance of dozing.

**Table A2:** Scores of STOP-BANG Sleep Apnea Questionnaire in post-COVID-19 patients.

		Yes	%
**S**	Do you snore loudly?	218	84.82%
**T**	Do you often feel tired, fatigued, or sleepy during the daytime?	154	59.92%
**O**	Has anyone observed you stop breathing or gasping during your sleep?	143	55.64%
**P**	Do you have, or are you being treated for, high blood pressure?	194	75.49%
**B**	BMI more than 35?	139	54.09%
**A**	Age over 50 years old?	180	70.04%
**N**	Neck circumference grater than 40 cm (male) or 37 cm (female)?	82	31.91%
**G**	Are you male?	171	66.54%
